# Lower dorsal striatum activation in association with neuroticism during the acceptance of unfair offers

**DOI:** 10.3758/s13415-015-0342-y

**Published:** 2015-02-27

**Authors:** Michelle Nadine Servaas, André Aleman, Jan-Bernard Cornelis Marsman, Remco Jan Renken, Harriëtte Riese, Johan Ormel

**Affiliations:** 1 Neuroimaging Center, Department of Neuroscience, University Medical Center Groningen, University of Groningen, PO Box 196, 9700 AD Groningen, The Netherlands; 2 Interdisciplinary Center Psychopathology and Emotion regulation (ICPE), Department of Psychiatry, University Medical Center Groningen, University of Groningen, Groningen, The Netherlands

**Keywords:** Functional magnetic resonance imaging (fMRI), Reward, Social decision-making, Ultimatum Game

## Abstract

Unfair treatment may evoke more negative emotions in individuals scoring higher on neuroticism, thereby possibly impacting their decision-making in these situations. To investigate the neural basis of social decision-making in these individuals, we examined interpersonal reactions to unfairness in the Ultimatum Game (UG). We measured brain activation with fMRI in 120 participants selected based on their neuroticism score, while they made decisions to accept or reject proposals that were either fair or unfair. The anterior insula and anterior cingulate cortex were more activated during the processing of unfair offers, consistent with prior UG studies. Furthermore, we found more activation in parietal and temporal regions for the two most common decisions (fair accept and unfair reject), involving areas related to perceptual decision-making. Conversely, during the decision to accept unfair offers, individuals recruited more frontal regions previously associated with decision-making and the implementation of reappraisal in the UG. High compared to low neurotic individuals did not show differential activation patterns during the proposal of unfair offers; however, they did show lower activation in the right dorsal striatum (putamen) during the acceptance of unfair offers. This brain region has been involved in the formation of stimulus–action–reward associations and motivation/arousal. In conclusion, the findings suggest that both high and low neurotic individuals recruit brain regions signaling social norm violations in response to unfair offers. However, when it comes to decision-making, it seems that neural circuitry related to reward and motivation is altered in individuals scoring higher on neuroticism, when accepting an unfair offer.

Neuroticism is one of the Big Five dimensions of personality (Costa & McCrae, 1989) and reflects individual differences in emotional reactivity, specifically in response to negative events (Canli, 2008). Individuals scoring higher on this personality trait tend to appraise events as more threatening and distressing than individuals scoring lower (“negativity bias”; Chan, Goodwin, & Harmer, 2007) and experience various negative emotional states more often and more intensely, such as depression and anxiety (Watson, Clark, & Harkness, 1994). High scores on neuroticism are considered an important risk marker for a variety of common mental disorders, in particular, internalizing disorders (Lahey, 2009; Ormel et al., 2013).

Epidemiological studies revealed that individuals with higher levels of neuroticism experience more stressful events and are emotionally more reactive to those events (Bolger & Schilling, 1991; Bolger & Zuckerman, 1995; Suls & Martin, 2005). Among the daily stressors investigated, interpersonal conflicts are reported more frequently by individuals scoring higher on neuroticism and cause the greatest amount of distress (Bolger & Schilling, 1991; Bolger & Zuckerman, 1995; Gunthert, Cohen, & Armeli, 1999). It has been argued that high neurotic individuals are emotionally more reactive because they tend to choose maladaptive interpersonal coping strategies (Gunthert et al., 1999), such as hostile reactivity (McCrae & Costa, 1986) and confrontation (Bolger & Zuckerman, 1995). In addition, these individuals display more avoidance and revenge motivations after an interpersonal conflict and are less forgiving (Brose, Rye, Lutz-Zois, & Ross, 2005; Maltby et al., 2008). These findings indicate that individuals scoring higher on neuroticism experience more emotional problems in dealing with interpersonal conflicts, which may impact their decision-making in these situations.

To investigate the neural processes involved in social decision-making in high neurotic individuals, we investigated reactions to unfairness in an “interactive” economic bargaining paradigm: the Ultimatum Game (UG; Sanfey, 2007). In short, one player (the proposer) suggests the division of a sum of money to another player (the responder). The responder has the option to accept the proposal, in which case money is divided according to the offer, or to reject the proposal, in which case neither player receives any money (Sanfey, Rilling, Aronson, Nystrom, & Cohen, 2003). According to economic models, one would expect the responder to accept any offer because even small earnings are preferable to none. To the contrary, however, prior research has shown that offers in which the proposer’s share exceeds 80 % of the total are rejected more than 50 % of the time (Camerer, 2003; Güth, Schmittberger, & Schwarze, 1982). This robust finding suggests that unfair treatment probably provokes negative emotions, such as anger, that causes individuals to punish their opponent at the expense of monetary reward (Fehr & Gächter, 2002). Possible reasons behind this seemingly irrational decision is to maintain a social reputation (Nowak, Page, & Sigmund, 2000) and/or to impose social norms in order to restore cooperation (Fehr & Gächter, 2002).

In line with this, neuroimaging research has shown the involvement of brain regions associated with fairness considerations, reward and emotion processing/regulation during the processing of unfair offers, such as the amygdala, striatum, anterior insula (AI), dorsal anterior cingulate cortex (dACC) and dorsolateral prefrontal cortex (dlPFC; Corradi-Dell’Acqua, Civai, Rumiati, & Fink, 2013; Gospic et al., 2011; Grecucci, Giorgetta, Van’t Wout, et al., 2013b; Guo, Zheng, Cheng et al., 2013; Guo, Zheng, Zhu et al., 2013; Güroğlu, van den Bos, Rombouts, & Crone, 2010; Güroğlu, van den Bos, van Dijk, Rombouts, & Crone, 2011; Harlé, Chang, van’t Wout, & Sanfey, 2012; Hollmann et al., 2011; Kirk, Downar, & Montague, 2011; Montague & Lohrenz, 2007; Sanfey et al., 2003; Zheng et al., 2014). Particularly, the latter three brain structures have been found consistently in research on the UG (Sanfey, 2007). Prior studies have shown that activation in the (i) AI is predictive of the rejection of unfair offers (Hollmann et al., 2011; Sanfey et al., 2003) and plays a key role in interoceptive awareness and the experience of subjective feelings (e.g., disgust and anger; Craig, 2009; Critchley, Wiens, Rotshtein, Ohman, & Dolan, 2004); (ii) dACC is involved in tracking error and conflict, evaluating the need for cognitive control and expectancy violation (Botvinick, Braver, Barch, Carter, & Cohen, 2001; Sanfey et al., 2003); (iii) dlPFC is implicated in executive control, inhibition and emotion regulation and remains fairly steady across unfair offers, possibly suggesting the maintenance of goal representations (e.g., maximizing monetary reward; Grecucci et al., 2013b; Petrides, 2005; Sanfey et al., 2003). A recent meta-analysis on the neural basis of social decision-making during the UG has shown robust activations in the (i) AI, ACC, medial supplementary motor area (mSMA) and cerebellum during unfair offers (> fair offers) and (ii) ACC, SMA and putamen during the rejection of unfair offers (> acceptance of unfair and fair offers; Gabay, Radua, Kempton, & Mehta, 2014).

Furthermore, Harlé et al. (2012) have found that sad mood, an emotion frequently experienced by individuals scoring higher on neuroticism (Watson et al., 1994), decreases the acceptance rates for unfair offers and influences activation in the AI, ACC and ventral striatum during these offers (Harlé et al., 2012). The authors proposed that sad individuals have an enhanced perception of social norm violations and show diminished sensitivity to rewarding social signals, such as the proposal of a fair offer (Harlé et al., 2012). Moreover, reappraisal, an emotion regulation strategy used less by high neurotic individuals (Gross & John, 2003), was associated with a larger number of unfair offers being accepted and increased activation in the dlPFC, ACC, medial PFC and temporoparietal areas in response to such offers (Grecucci et al., 2013b). It was suggested that individuals attempt to cognitively modulate their negative emotions by reinterpreting the intentions of their opponents in order to overcome emotional motivations (e.g., punishment) and make a rational decision (i.e., accept unfair offers; Grecucci et al., 2013b).

The aim of the current study was to investigate the association between neuroticism and brain activation during the perception of social norm violations and social decision-making in the UG, specifically in response to unfair offers. We implemented an adapted form of the UG (Sanfey et al., 2003) in a sample of 120 women selected on the basis of their neuroticism score. Only women were included, because they tend to significantly score higher on neuroticism than men and have a higher risk of developing affective disorders (Parker & Brotchie, 2010). Furthermore, research is still limited related to gender differences in neuroticism. Therefore, we decided not to introduce this source of possible variation in the sample, as it is not properly understood yet. First, we hypothesized increased rejection rates of unfair offers to be associated with higher levels of neuroticism. Second, we hypothesized differential activation in brain regions related to fairness considerations (increased activation), reward (increased [unfair reject]/decreased [unfair accept] activation) and the processing and regulation of negative affect (increased activation) (e.g., AI, dACC, dlPFC, striatum) in individuals scoring higher on neuroticism during the proposal of unfair offers and the decision to reject/accept them. Both hypotheses were based on the studies of Harlé et al. (2012) and Grecucci et al. (2013b).

## Method

### Participants

Initially, 240 students from the University of Groningen were asked to fill out the NEO Five-Factor Inventory (NEO-FFI; domains Neuroticism and Extraversion, 24 items). Individuals were sent a questionnaire when they agreed to participate in the study (based on the information letter, which included an informed consent form) and met the following selection criteria: (1) female gender, (2) age between 18–25 years, (3) Dutch as native language, (4) Caucasian descent, (5) right handed, (6) no use of contraceptive medication, except for oral contraceptive pills (21-pill packet). Exclusion criteria were (1) a history of seizure or head injury, (2) a lifetime diagnosis of psychiatric and/or neurological disorders, (3) a lifetime diagnosis of psychiatric disorders in first-degree relatives of the participant, (4) the use of medication that can influence test results, (5) visual or auditory problems that cannot be corrected, (6) MRI incompatible implants or tattoos, (7) claustrophobia, (8) suspected or confirmed pregnancy. We selected a homogeneous sample, using the former set of specific and narrow criteria, in order to control for possible confounding influences due to gender, age, education level and ethnicity, thereby increasing our power. From this sample, 120 individuals (mean age: 20.8 *SD* ± 2.0, age range: 18–25) were invited to participate in the experiment. To ensure sufficient numbers of participants with high levels of neuroticism, 60 individuals were selected from the highest quartile of neuroticism scores (NEO-FFI score ≥ 32, range 32–47) and 60 individuals were randomly selected from the three lowest quartiles (NEO-FFI < 32, range 17–31). Plots of normality (QQ-plot and boxplot) showed that, in the selected 120 participants, neuroticism scores were approximately normally distributed. The sample size of the current study was based on the fact that we also genotyped two polymorphisms (5-HTTLPR and COMT) and aimed to have sufficient numbers of individuals in each genetic group (>25), considering the allele frequencies in the general population (results are not reported in this manuscript). The reason that we only selected individuals of Caucasian descent is that genetic architecture has been shown to be different between ethnicities (Munafò et al., 2003).

In order to reduce hormone-related between-subject variability, participants were invited for the experiment during the first 10 days of their menstrual cycle (early and mid-follicular phase) or during the discontinuation week in case of oral contraceptive usage, which resembles the early and mid-follicular phase in terms of ovarian hormonal levels (Cohen & Katz, 1979). During these phases, ovarian hormonal levels are relatively low and menstrual-cycle related changes in mood, stress sensitivity and neurocognitive function are minimal (Andreano & Cahill, 2010; Goldstein, Jerram, Abbs, Whitfield-Gabrieli, & Makris, 2010; Symonds, Gallagher, Thompson, & Young, 2004).

On the day of the experiment, after explaining the procedure, participants gave informed consent again and completed the NEO Personality Inventory Revised (NEO-PI-R; domains Neuroticism, Extraversion and Conscientiousness, 144 items; Hoekstra, Ormel, & De Fruyt, 1996). The study was approved by the Medical Ethical Committee of the University Medical Center Groningen and was conducted in accordance with the Declaration of Helsinki.

### Experimental design

**Fig. 1:**
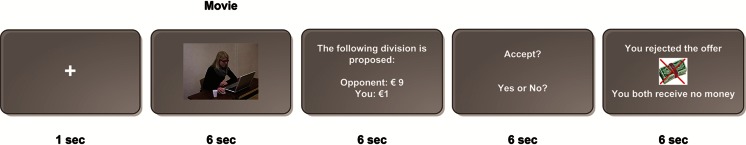
Task outline. First, participants were presented with a fixation cross (1 sec). Second, a movie clip was played in which participants could observe their opponent making a decision behind a computer (6 sec). Third, a fair proposal (€5:€5) or an unfair proposal (€9:€1, €8:€2, and €7:€3) was randomly presented (6 sec). Fourth, participants were able to accept or reject the proposal (6 sec). Fifth, the outcome was presented, that is, the amount of money that each participant earned for that particular trial

Participants acted as responders in a series of 24 trials of the UG, wherein splits of €10 were proposed. A trial consisted of the following five subcomponents. First, participants were presented with a fixation cross for one second. Second, a movie clip was played in which participants observed a female opponent (the same for every trial) sitting behind a computer screen and moving the mouse with her right hand in order to propose a division. We presented movie clips instead of pictures, to make the design more ecologically valid. The movie clips were played in a serial order and had a duration of six seconds (see Appendix A for more details on the movie clips). Third, participants were randomly presented with either a fair proposal (€5:€5) or an unfair proposal (€9:€1, €8:€2 and €7:€3) for six seconds. Fourth, participants were able to accept or reject the proposal during a six-second time window. When participants accepted the proposal, money was divided according to the offer. However, when participants rejected the proposal, both players did not receive any money. Participants were instructed to press the left button on the button box to accept the proposal and the second button on the left to reject the proposal. Fifth, the outcome was shown, that is, the amount of money that each participant earned for that particular trial. The outcome screen had a duration of six seconds, after which a new trial started. Participants were told that they would be paid a percentage (10 %) of the money they had earned during the game, in addition to a fixed amount for their participation in the experiment. However, all participants received the same amount of money due to guidelines from the local ethical committee. In total, four blocks were presented, including the following offer rates per block: 3 × (€5:€5), 1 × (€9:€1), 1 × (€8:€2) and 1 × (€7:€3). Rest periods with a duration of 15 seconds, in which a fixation cross was shown, were presented at the beginning of the task, the end of the task and in between the four blocks. The duration of a trial was 25 seconds, and the total duration of the experimental paradigm was 11.7 minutes (see Fig. 1 for the task outline and Appendix B for an overview of the full fMRI session).

### Image acquisition

A 3 Tesla Philips Intera MRI scanner (Philips Medical Systems, Best, the Netherlands), equipped with a 32-channel SENSE head coil, was used to acquire the images. A high-resolution T1-weighted 3D structural image was obtained using fast-field echo (FFE) for anatomical reference (170 slices; TR: 9 ms; TE: 8 ms; FOV: 256 × 231; 256 × 256 matrix; voxel size: 1 × 1 × 1 mm). Functional images were acquired with T2*-weighted gradient echo planar imaging (EPI) sequences. The experimental paradigm comprised 351 volumes of 39 axial-slices (TR: 2000 ms; TE: 30 ms; FOV: 224 × 224; 64 × 61 matrix; voxel size: 3.5 × 3.5 × 3.5 mm). Slices were acquired in descending order without a gap. To prevent artifacts due to nasal cavities, images were tilted 10° to the AC-PC transverse plane.

### Statistical analyses

#### Questionnaire and behavioral analysis

Behavioral analyses were performed using IBM SPSS Statistics Version 20 (IBM, SPSS Inc., Chicago, IL, USA). Percentages of rejection as well as acceptance were calculated for unfair and fair offers, respectively. We also calculated the percentages of rejection for the three different types of unfair offers separately (€9:€1; €8:€2; €7:€3). Furthermore, due to nonnormality of the data, a Friedman’s ANOVA was performed to investigate the main effect of offer amount. Post hoc analyses were performed with a Wilcoxon signed-rank test. In addition, a Spearman’s correlation was calculated between NEO-PI-R neuroticism scores and rejection/acceptance rates of unfair and fair offers. Behavioral results with *p* values < .05 were considered significant.

#### Image analysis

Image processing and statistical analyses were performed using SPM8 (http://www.fil.ion.ucl.ac.uk/spm), implemented in Matlab 7.8.0 (The Mathworks Inc., Natick, MA). Preprocessing included realignment, coregistration, DARTEL normalization (2 mm^3^ isotropic voxels; Ashburner, 2007) and smoothing (8-mm full-width at half maximum [FWHM] Gaussian kernel; see Appendix C for details on the preprocessing steps). Six subjects were excluded from further analysis; two because of anatomical abnormalities (i.e., large ventricles that were still within the normal range but difficult to normalize) and four because of technical problems with the scanner or task computer. A total sample of 114 subjects remained.

Hemodynamic changes for each condition were calculated using the General Linear Model (GLM). In the GLM, predictors were created for the following subcomponents of each trial with their respective duration between brackets: introduction rest period (2 s), movie (6 s), proposal fair (6 s), proposal unfair (6 s), decision fair accepted (6 s), decision unfair accepted (6 s), decision fair rejected (6 s), decision unfair rejected (6 s), outcome fair accepted (6 s), outcome unfair accepted (6 s), outcome fair rejected (6 s), outcome unfair rejected (6 s). Effects were modeled using a boxcar function convolved with the canonical hemodynamic response function (HRF). Furthermore, six rigid-body head motion parameters, their first temporal derivatives and a constant term (overall signal mean) were included in the design matrix. The following contrasts were computed per subject on first level: (proposal unfair vs proposal fair), (decision unfair rejected vs decision fair accepted) and (decision unfair accepted vs decision fair accepted).

For the proposal condition, the resulting contrast images (unfair vs fair; within-subject factor) were entered in a second-level random-effect analysis. Neuroticism scores were mean centered and entered as a regressor of interest in the model (between-subject factor). Main effects as well as interactions with neuroticism (i.e., investigating whether the slope of the association between neuroticism and brain activation was significantly different for the different task conditions) were investigated using *t* contrasts in SPM. To correct for multiple comparisons, resulting brain images were thresholded at *p* < .05 FWE cluster-level extent using an initial threshold of *p* < .001 uncorrected. Furthermore, rejection rates for unfair proposals were mean centered and entered as a regressor of interest in the model for the contrast (unfair > fair) using SnPM5 (http://warwick.ac.uk/snpm; MultiSub, simple regression) to investigate whether certain brain regions predict the subsequent decision to reject an unfair offer (Sanfey et al., 2003). We used SnPM because rejection rates for unfair proposals were not normally distributed. To correct for multiple comparisons, resulting brain images were thresholded at *p* < .05 FWE.

For the decision condition, the resulting contrast images (unfair rejected vs fair accepted, and unfair accepted vs fair accepted) were entered in a second-level two-way ANOVA (factor 1: unfairness, 2 levels: fair, unfair; factor 2 response, 2 levels: reject, accept) subsumed in a linear-mixed effects (LME) framework (*3dLME*, implemented in AFNI, http://afni.nimh.nih.gov/afni/; for details on the method, see Chen, Saad, Britton, Pine, & Cox, 2013). Analyses were performed within this framework because the design is unbalanced due to missing data. Specifically, the number of conditions varied across subjects, because different choices could be made during the game, e.g., only 24 subjects rejected one or more fair proposals (for details on the number of subjects per decision type, see Table 1). Neuroticism was mean centered and entered as a regressor of interest in the model. We examined the following contrasts (unfair rejected vs fair accepted), (unfair accepted vs fair accepted) and (unfair rejected vs unfair accepted) and their interactions with neuroticism. To correct for multiple comparisons, resulting brain images were thresholded at *p* < .05 FWE cluster-level extent using an initial threshold of *p* < .001 uncorrected and a cluster size of *k* > 102. The extent threshold (*k*) was obtained via Monte Carlo simulation (*3dClustSim*, AlphaSim, implemented in AFNI, 10,000 iterations).

**Table 1:** Number of subjects per decision type

	Proposal fair	Proposal unfair
Decision reject	24	107
Decision accept	113	71

## Results

### Questionnaire and behavioral data

The mean NEO-PI-R neuroticism score across the whole sample was 135.47 ± *SD* 18.92 (range: 94–195), which is comparable to the mean of the Dutch female student norm group in the NEO-manual (*n* = 690, 143.6 ± 21.0; Hoekstra et al., 1996). Furthermore, behavioral results showed that 73.6 % of the unfair offers were rejected and 94.9 % of the fair offers were accepted. Rejection rates for the three different types of unfair offers were 88.1 % for offers of €9:€1, 75.8 % for offers of €8:€2 and 56.7 % for offers of €7:€3. In addition, the main effect of offer amount was found to be significant, χ^2^ (3) = 203.31, *p* < .0001). Post hoc analyses revealed that (i) unfair offers of €9:€1 (mean: 3.51, *SD* ± 1.12) were significantly more rejected than unfair offers of €8:€2 (mean: 3.02, *SD* ± 1.48; *Z* = 5.12, *p* < .0001), (ii) unfair offers of €8:€2 were significantly more rejected than unfair offers of €7:€3 (mean: 2.23, *SD* ± 1.65; Z = 5.75, *p* < .0001) and (iii) unfair offers of €7:€3 were significantly more rejected than fair offers of €5:5 (mean: 0.61, *SD* ± 1.94; Z = 6.56, *p* < .0001). Moreover, we found no significant correlation between NEO-PI-R neuroticism scores and rejection/acceptance rates of unfair and fair offers (*p* > .17).

### Imaging data

#### Main effects of the proposal condition

**Fig. 2:**
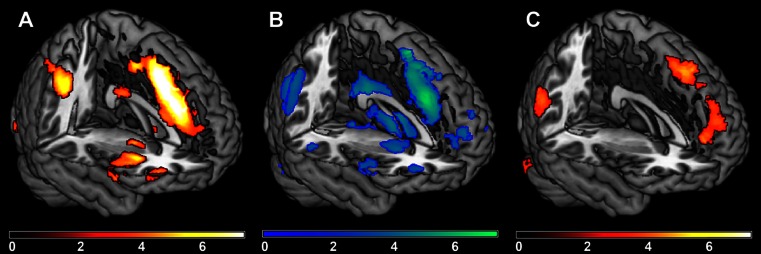
Main effects of the proposal and decision condition, and the interaction effect between rejection rates and the proposal condition. **a**. Brain regions that showed higher activation for the contrast (proposal unfair > proposal fair). **b**. Brain regions that correlated negatively with rejection rates for unfair proposals for the contrast (proposal unfair > proposal fair). **c**. Brain regions that showed higher activation for the contrast (decision unfair accepted > decision fair accepted)

Brain regions were identified for the contrast (unfair vs fair). We found higher activation in the anterior insula, dorsal anterior cingulate cortex, (dorso)lateral prefrontal cortex, inferior/superior parietal gyrus, cerebellum, thalamus and pallidum during unfair proposals compared to fair proposals (see Table 2 and Fig. 2a for the results). For the reverse contrast (fair > unfair), we found higher activation in the precuneus, middle cingulate gyrus, superior temporal gyrus and precentral gyrus (see Table 2 for the results).

**Table 2:** Main effects of the proposal condition

Cluster	Cluster size	*T* value	*Z* value	Coordinates		
				*x*	*y*	*z*
Unfair > fair						
Anterior cingulate Middle cingulate Superior medial frontal gyrus Supplementary motor area	4224	7.80	6.95	8	18	44
		7.79	6.95	6	26	34
		7.72	6.90	-2	18	46
Postcentral gyrus Inferior parietal gyrus Superior parietal gyrus	3745	7.15	6.48	-44	-28	48
		6.96	6.33	-34	-44	48
		6.83	6.23	-34	-24	52
Cerebellum Inferior occipital gyrus Fusiform gyrus	1178	6.68	6.11	-40	-62	-32
		5.67	5.30	-36	-64	-44
		4.00	3.86	-26	-82	-46
Cerebellum Vermis Inferior occipital gyrus Fusiform gyrus	3385	6.39	5.88	18	-52	-24
		6.33	5.84	32	-56	-30
		5.92	5.51	10	-56	-16
Insula Inferior frontal gyrus pars orbitalis Superior temporal pole	865	6.28	5.80	-40	14	-6
		5.99	5.57	-28	16	-10
Angular gyrus Supramarginal gyrus Inferior parietal gyrus Superior parietal gyrus Middle occipital gyrus Superior occipital gyrus	1702	6.16	5.71	28	-60	48
				44	-36	38
				36	-44	38
Insula Inferior frontal gyrus Superior temporal pole	830	5.54	5.20	36	24	-4
		5.19	4.90	36	14	-4
		4.95	4.70	44	20	-6
Thalamus Pallidum Amygdala Hippocampus	561	5.12	4.85	-12	-20	2
		4.23	4.06	8	-12	-12
		4.09	3.94	-14	-8	-10
Precentral gyrus Inferior frontal gyrus pars opercularis	348	4.77	4.54	-44	2	32
		3.83	3.70	-32	4	26
		3.81	3.69	-56	8	30
Middle frontal gyrus Inferior frontal gyrus pars triangularis Inferior frontal gyrus pars opercularis Precentral gyrus	1171	4.73	4.51	42	36	18
		4.53	4.33	38	30	26
		4.51	4.31	38	48	12
Pallidum Caudate	272	4.69	4.47	14	4	-4
		4.32	4.14	16	12	8
		3.69	3.57	18	-2	4
Fair > unfair						
Precuneus Middle cingulate gyrus Supramarginal gyrus Calcarine sulcus Superior temporal gyrus Rolandic operculum	3814	5.82	5.42	12	-54	16
		5.15	4.87	56	-32	22
		5.13	4.85	52	-6	12
Precentral gyrus Postcentral gyrus	597	5.34	5.03	38	-18	50

Peak activations with corresponding *T* values and *Z* values of brain regions, which showed differential activation for the contrast (unfair vs fair). To correct for multiple comparisons, resulting brain images were thresholded at *p* < .05 FWE cluster-level extent using an initial threshold of *p* < .001 uncorrected

#### Interaction effect between rejection rates and the proposal condition

Brain regions were identified that correlated with rejection rates for unfair proposals for the contrast (unfair > fair). Rejection rates for unfair proposals were associated with lower activation in cingulate and frontal regions, the supplementary motor area, angular gyrus, inferior parietal gyrus and cerebellum during unfair proposals compared to fair proposals (see Table 3 and Fig. 2b for the results).

**Table 3:** Interaction effect between rejection rates and the proposal condition

Cluster	Cluster size	Pseudo *T* value	Coordinates		
			*x*	*y*	*z*
Unfair > fair * rejection rates for unfair proposals					
Superior frontal gyrus Supplementary motor area	54	6.47	-14	12	60
		6.34	-10	6	66
		4.80	-2	10	62
Middle frontal gyrus Precentral gyrus	284	6.47	-46	14	42
		6.14	-42	24	40
Anterior cingulate gyrus Middle cingulate gyrus Superior medial frontal gyrus Supplementary motor area	352	6.39	0	28	36
		5.27	0	16	52
		5.24	-2	20	60
Inferior frontal gyrus	156	6.24	-50	18	-4
Cerebellum	26	5.65	30	-84	-36
		4.82	36	-80	-40
Inferior frontal gyrus pars triangularis Middle frontal gyrus	26	5.16	-46	46	4
Cerebellum	34	5.12	8	-82	-28
Angular gyrus Inferior parietal gyrus	36	5.00	-44	-60	36
Supplementary motor area	4	4.96	14	16	62
Middle cingulate gyrus	8	4.86	0	-28	28
Middle frontal gyrus	5	4.84	38	38	34

Peak activations with corresponding pseudo *T* values of brain regions, which showed differential activation for the contrast (unfair > fair) * rejection rates of unfair proposals. To correct for multiple comparisons, resulting brain images were thresholded at *p* < .05 FWE

#### Main effects of the decision condition

First, brain regions were identified for the contrast (unfair rejected vs fair accepted). We found higher activation in the lingual gyrus during the acceptance of fair proposals compared to the rejection of unfair proposals (see Table 4 for the results). No significant results were observed for the reverse contrast (unfair rejected > fair accepted). Second, brain regions were identified for the contrast (unfair accepted vs fair accepted). We found higher activation in the anterior cingulate gyrus, superior (medial) frontal gyrus, supplementary motor area, angular gyrus and cerebellum during the acceptance of unfair proposals compared to the acceptance of fair proposals (see Table 4 and Fig. 2c for the results). For the reverse contrast (fair accepted > unfair accepted), we found higher activation in the inferior/superior parietal gyrus, precuneus, occipital gyrus, inferior/middle temporal gyrus, fusiform gyrus, insula, hippocampus and caudate (see Table 4 for the results). Fourth, brain regions were identified for the contrast (unfair rejected vs unfair accepted). We found higher activation in the inferior/superior parietal gyrus, precuneus, occipital gyrus, inferior/middle temporal gyrus, hippocampus and thalamus during the rejection of unfair proposals compared to the acceptance of unfair proposals (see Table 4 for the results). No significant results were observed for the reverse contrast (unfair accepted > unfair rejected).

**Table 4:** Main effects of the decision condition and interaction effects between neuroticism and the decision condition

Cluster	Cluster size	*Z* value	Coordinates		
			*x*	*y*	*z*
Fair accepted > unfair rejected					
Lingual gyrus	127	3.66	-14	-74	-6
Unfair accepted > fair accepted					
Cerebellum	279	5.07	28	-88	-38
Cerebellum	293	5.17	-36	-84	-42
Anterior cingulate gyrus Superior medial frontal gyrus	418	4.87	4	54	14
Angular gyrus	179	4.32	60	-52	34
Angular gyrus Supramarginal gyrus	144	4.77	-62	-54	32
Superior medial frontal gyrus Superior frontal gyrus Supplementary motor area	1118	5.34	12	18	60
Fair accepted > unfair accepted					
Inferior occipital gyrus Middle occipital gyrus Inferior temporal gyrus Middle temporal gyrus Fusiform gyrus	1,197	6.71	-38	-64	-2
Inferior occipital gyrus Inferior temporal gyrus Middle temporal gyrus Fusiform gyrus	658	5.18	50	-58	-6
Insula Superior temporal gyrus	113	3.93	-44	0	-10
Hippocampus Caudate	134	3.73	-30	-40	-6
Hippocampus Caudate	159	4.06	22	-34	12
Superior parietal gyrus Precuneus Angular gyrus Superior occipital gyrus	1789	5.48	18	-62	62
Inferior parietal gyrus Superior parietal gyrus Precuneus Postcentral gyrus Middle occipital gyrus Superior occipital gyrus	3217	5.81	-20	-58	54
Postcentral gyrus Supramarginal gyrus	488	5.20	36	-38	38
Unfair rejected > unfair accepted					
Inferior temporal gyrus Middle temporal gyrus	225	4.95	52	-58	-4
Inferior occipital gyrus Middle occipital gyrus Inferior temporal gyrus Middle temporal gyrus	422	5.06	-38	-64	-2
Hippocampus Thalamus	112	3.85	-4	-26	14
Superior parietal gyrus Precuneus Angular gyrus Postcentral gyrus Middle occipital gyrus Superior occipital gyrus	1540	5.29	24	-66	44
Postcentral gyrus Supramarginal gyrus	227	4.43	40	-38	42
Inferior parietal gyrus Superior parietal gyrus Precuneus Postcentral gyrus	3730	5.22	-22	-56	68
Unfair accepted > fair accepted * neuroticism					
Vermis Cerebellum	381	-4.31	-10	-42	-20
Putamen	307	-5.44	28	-8	12
Unfair accepted > unfair rejected * neuroticism					
Putamen	282	-5.30	28	-10	12

Peak activations with corresponding *Z* values of brain regions, which showed differential activation for the contrasts (fair accepted > unfair rejected), (unfair accepted vs fair accepted), (unfair rejected > unfair accepted), (unfair accepted > fair accepted) * neuroticism and (unfair accepted > unfair rejected) * neuroticism. To correct for multiple comparisons, resulting brain images were thresholded at *p* < .05 FWE cluster level extent using an initial threshold of *p* < .001 uncorrected and a cluster size of *k* > 102. The extent threshold (*k*) was obtained via Monte Carlo simulation (*3dClustSim*, AlphaSim, implemented in AFNI, 10,000 iterations)

#### Interaction effect between neuroticism and the proposal/decision condition

**Fig. 3:**
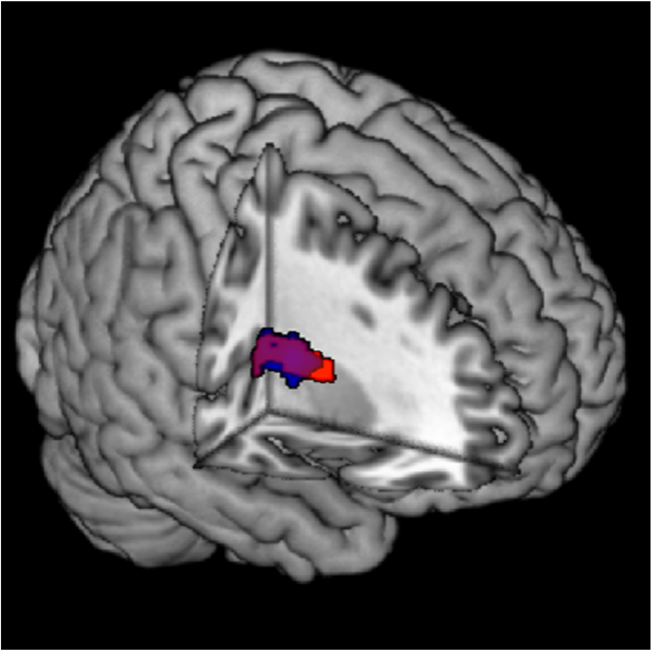
Interaction effect between neuroticism and the decision condition. Activation in the dorsal striatum correlated negatively with neuroticism for the contrast (decision unfair accepted > decision fair accepted) (red) and (decision unfair accepted > decision unfair rejected) (blue). The color purple indicates overlap between the two contrasts

Brain regions were identified that correlated with neuroticism for abovementioned contrasts. Neuroticism was associated with lower activation in (i) the dorsal striatum (putamen) and vermis/cerebellum for the contrast (unfair accepted > fair accepted) and (ii) the dorsal striatum (putamen) for the contrast (unfair accepted > unfair rejected; see Table 4 and Fig. 3 for the results). No significant results were found for the other contrasts. When the results were visualized in a scatter plot, we observed a negative correlation between neuroticism and activation in the dorsal striatum for the condition unfair accepted (unfair accepted > fair accepted *r* = -0.40; unfair accepted > unfair rejected *r* = -0.48), while a weak correlation was observed for the conditions fair accepted (*r* = 0.04) and unfair rejected (*r* = 0.14; see Appendix D for scatter plots). For a complete overview, results for the interaction effect between neuroticism and the proposal (thresholded at *p* < .05 FWE cluster-level extent using an initial threshold of *p* < .01 uncorrected), decision (thresholded at *p* < .05 FWE cluster-level extent using an initial threshold of *p* < .01 uncorrected and a cluster size of *k* > 316) and outcome (thresholded at *p* < .05 FWE cluster-level extent using an initial threshold of *p* < .001 and *p* < .01 uncorrected and a cluster size of *k* > 102 and *k* > 316, respectively) condition can be found in Appendix E, Table 6.

## Discussion

The aim of the current study was to investigate the relationship between neuroticism and brain activation during the perception of social norm violations and social decision-making in the UG, specifically in response to unfair offers. We observed higher activation in brain regions that have previously been found in research on the UG during unfair proposals, including the anterior insula, dorsal anterior cingulate cortex and dorsal lateral prefrontal cortex (Sanfey et al., 2003). Furthermore, we found more activation in parietal and temporal regions for the two most common decisions (fair accept and unfair reject), involving areas related to perceptual decision-making (Heekeren, Marrett, & Ungerleider, 2008; Keuken et al., 2014). Conversely, during the decision to accept unfair offers, individuals recruited more frontal regions previously associated with decision-making and the implementation of reappraisal in the UG (Grecucci et al., 2013b). Individuals scoring higher on neuroticism did not show differential activation patterns during the proposal of unfair offers compared with individuals scoring lower; however, they did show lower activation in the right dorsal striatum (putamen) during the acceptance of unfair offers. Activation in the dorsal striatum has been implicated in the formation of stimulus–action–reward associations (Balleine, Delgado, & Hikosaka, 2007; FitzGerald, Friston, & Dolan, 2012; Haruno & Kawato, 2006; Peterson & Seger, 2013) and motivation and arousal (Miller, Shankar, Knutson, & McClure, 2014; Takeuchi et al., 2014). The findings suggest that both high and low neurotic individuals recruit brain regions signaling social norm violations in response to unfair offers. However, when it comes to decision-making, it seems that neural circuitry related to reward and motivation is altered in individuals scoring higher on neuroticism, when accepting an unfair offer.

### Results related to the proposal condition

Our findings replicated and confirmed previous behavioral as well as imaging results. First, increased rejection rates were found as offers became more unfair. Second, we found more activation in brain regions that have been consistently found in studies on the UG during unfair proposals relative to fair proposals, such as the AI, dACC and (d)lPFC (Corradi-Dell’Acqua et al., 2013; Gospic et al., 2011; Grecucci et al., 2013b; Guo, Zheng, Cheng et al., 2013; Guo, Zheng, Zhu et al., 2013; Güroğlu et al., 2010; Güroğlu et al., 2011; Harlé et al., 2012; Hollmann et al., 2011; Kirk et al., 2011; Montague & Lohrenz, 2007; Sanfey et al., 2003; Zheng et al., 2014). These results confirm the findings of aforementioned previous studies, suggesting that this network —known for its involvement in “hot cognition” or emotional aspects affecting cognition (Ochsner & Gross, 2005)—subserves responses to social norm violations (for a computational rendering of the underlying neural substrates of social norm violations, see Xiang, Lohrenz, & Montague, 2013). Converging evidence for the role of emotional states during social norm violations and social decision-making in the UG comes from, among others, behavioral and skin conductance studies that revealed more reported negative emotions (e.g., disgust and anger) in response to unfair offers (Pillutla & Murnighan, 1996; Xiao & Houser, 2005) and higher skin conductance activity to unfair offers, which correlated with the number of subsequent rejections (van’t Wout, Kahn, Sanfey, & Aleman, 2006).

### Results related to the decision condition

For the two most common decisions (fair accept and unfair reject), we found more activation in several parietal, temporal and occipital brain regions as well as the insula, hippocampus and caudate. These regions have been implicated in perceptual decision-making (for a meta-analysis, see Keuken et al., 2014), which consists of multiple subprocesses. For example, the representation of sensory evidence (e.g., occipital regions and hippocampus), the detection of perceptual uncertainty or difficulty (e.g., insula) and the distribution of attentional resources (e.g., parietal regions; for a review, see Heekeren et al., 2008). However, during the decision to accept unfair offers, individuals recruited more frontal regions (e.g., the anterior cingulate cortex and dorsal [medial] prefrontal cortex), which have been associated with the computation of decision variables and the monitoring of performance during perceptual decision-making (Heekeren et al., 2008; Keuken et al., 2014). Furthermore, they have been related to the implementation of reappraisal strategies in response to unfair offers during the UG (Grecucci et al., 2013b). Thus, these frontal regions may be involved in cognitive control and the regulation of negative emotions in order to make a rational decision and gain monetary reward (Grecucci & Sanfey, 2013; Grecucci, Giorgetta, Bonini, et al., 2013a; Grecucci et al., 2013b). Earlier studies have shown that, during the UG, less unfair offers were rejected when individuals used reappraisal compared to other regulation strategies, such as expressive suppression (Grecucci et al., 2013b; van’t Wout et al., 2010). In line with this, we found more activation in cingulate and frontal brain regions during the proposal of unfair offers, when individuals subsequently rejected less of them.

### Results related to neuroticism

Individuals scoring higher on neuroticism did not reject more unfair offers than individuals scoring lower, neither did they show differences in brain activation during the proposal of unfair offers. This may indicate that both high and low neurotic individuals perceive unfair offers as social norm violations that are unjust, i.e., rejecting them most of the time and recruiting brain regions related to social norm violations (e.g., AI, dACC and dlPFC; Sanfey et al., 2003; Xiang et al., 2013). However, we did observe lower activation in the right dorsal striatum (putamen) in individuals with higher scores on neuroticism during the acceptance of unfair offers compared to the acceptance of fair offers and the rejection of unfair offers.

Two functions of the dorsal striatum (putamen) may be of relevance to the current findings. First, it has been involved in the formation of stimulus–action–reward associations (Balleine et al., 2007; FitzGerald et al., 2012; Haruno & Kawato, 2006; Peterson & Seger, 2013). Specifically, the dorsal striatum assigns values to specific actions, which are then weighted against each other in order to direct adaptive decision-making (FitzGerald et al., 2012). A study—that isolated brain activation in subregions of the striatum during different intratrial processes (stimulus, preparation of response, response and feedback) in a visuomotor learning task—showed that the putamen was active during all processes, but to a higher degree during response (Peterson & Seger, 2013). Furthermore, during stimulus presentation, activation in the putamen was related to the magnitude of the upcoming reward (Peterson & Seger, 2013). It was proposed that the putamen may be involved in policy selection on the basis of relative preferences between actions (Haruno & Kawato, 2006; Peterson & Seger, 2013). Second, the dorsal striatum has been implicated in motivation and arousal (Miller et al., 2014; Takeuchi et al., 2014). Related to the function of forming stimulus–action–reward associations, motivation refers to the drive for action to obtain rewards or to avoid punishments and includes the preparation and planning of actions to realize such goals (Miller et al., 2014). In addition, dorsal striatum activation has been related to competitive and academic achievement motivation and monetary motivation (Mizuno et al., 2008; Takeuchi et al., 2014). In conclusion, it is possible that individuals scoring higher on neuroticism are less motivated and/or experience less feelings of reward, when they decide to accept unfair offers. This is in line with the finding of Harlé et al. (2012) that individuals in a sad mood, an emotion frequently experienced by individuals scoring higher on neuroticism (Watson et al., 1994), showed diminished sensitivity to rewarding offers in the UG in comparison to individuals in a neutral state. Notably, this tendency of reduced motivation to obtain rewards or ability to experience rewards has also been observed in depression (Groenewold, Opmeer, de Jonge, Aleman, & Costafreda, 2013; Zhang, Chang, Guo, Zhang, & Wang, 2013), for which neuroticism is a potent risk factor (Lahey, 2009; Ormel et al., 2013). However, caution is needed with the interpretation of these results since other functions of the putamen cannot be ruled out, such as response-related functions (e.g., selection and working-memory; Peterson & Seger, 2013).

The fact that we found differences in brain activation during the decision phase, but not during the proposal phase, may indicate that differences associated with neuroticism may be more related to the recovery phase than the initial response to unfair offers. This effect in temporal dynamics has also been found in relation to the presentation of negative images in individuals scoring higher on neuroticism (Schuyler et al., 2014). We may speculate that negative affect lingers longer in individuals scoring higher on neuroticism because they apply maladaptive coping strategies and for this reason experience less reward, while accepting unfair offers. However, this should be confirmed in future research. Besides investigating the temporal dynamics, it would be of interest to investigate functional connectivity patterns related to neuroticism during social decision-making in the UG, since previous studies have found altered connectivity in relation to neuroticism (Bjørnebekk et al., 2013; Cremers et al., 2010; Servaas et al., 2013; Servaas et al., 2014; Xu & Potenza, 2012)

### Limitations

Several limitations can be mentioned with regard to the current study. First, no affective ratings and autonomic measures were collected during the experiment. Such ratings and measures could have provided converging evidence for abovementioned interpretations, which are at present speculative. Second, it is possible that we would have found an association between neuroticism and rejection/acceptance rates or brain activation during the proposal phase, when we had included a greater range of offers (e.g., €10:€10–€19:€1; Kirk et al., 2011). The reason for this may be that high and low neurotic individuals make similar decisions for offers on the extremes, but different ones for offers in the middle (i.e., the “grey” area). Third, we did not have an extensive cover story while introducing the task. Participants were told that they were going to play a game in the scanner against an opponent, who offers them splits of €10 that they subsequently can accept or reject. No specific information was provided about the opponent, the way she was selected or her knowledge about certain aspects of the game. It is possible that this has affected the credibility of the task. However, effects in the UG are quite robust and task effects, which have been found in previous UG studies, were replicated in our study. Fourth, we were unable to tear apart the effects related to fairness and magnitude of the offer (i.e., did participants accept more [€5:€5] offers because they were fair, or simply because it represented more money?). It may be better to use offers with varying percentages of different stakes sizes (e.g., 20 %–33 % of the stake [5 or 15 tokens], Vieira et al., 2014). Fifth, we note that the number of accepted unfair offers may be too small, since only 26.4 % of the unfair offers were accepted. Even though we have a large sample size (the unfair accept condition was present, at least once, in 71 subjects) and subsumed our model in a LME framework (which is particularly suitable for these type of data), this may have lowered the power to detect possible effects. Sixth, this task was part of a larger experimental protocol (see Appendix B for an overview of the full fMRI session). Conceivably, preceding tasks may have had an effect on the data described here.

## Conclusion

Whereas no relationship between neuroticism and brain activation during the proposal of unfair offers was observed, our results showed lower activation in the dorsal striatum (putamen) in individuals scoring higher on neuroticism during the acceptance of unfair offers (i.e., decision phase). Activation in the dorsal striatum has been implicated in the formation of stimulus–action–reward associations (Balleine et al., 2007; FitzGerald et al., 2012; Haruno & Kawato, 2006; Peterson & Seger, 2013) and motivation and arousal (Miller et al., 2014; Takeuchi et al., 2014). The findings suggest that both high and low neurotic individuals recruit brain regions signaling social norm violations in response to unfair offers. However, when it comes to decision-making, neural circuitry related to reward and motivation may be altered in individuals scoring higher on neuroticism, when accepting an unfair offer. It would be of interest for future studies to investigate how negative affect in response to unfairness, possibly due to maladaptive coping, has an effect on the ability to experience reward during subsequent decision-making.

## Figures and Tables

**Fig. 4:**
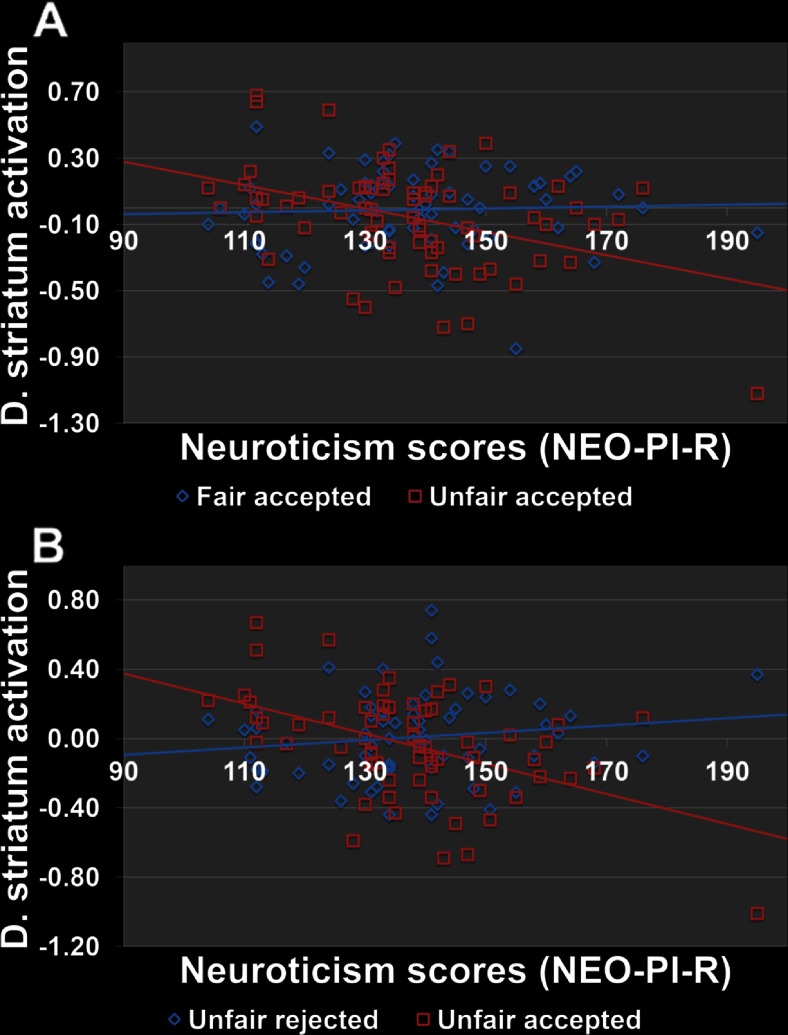
Effects related to neuroticism. A. Interaction effect between neuroticism and the decision condition (contrast unfair accepted > fair accepted) on dorsal striatum activation. B. Interaction effect between neuroticism and the decision condition (contrast unfair accepted > unfair rejected) on dorsal striatum activation. A negative correlation was found between neuroticism and activation in the dorsal striatum for the condition unfair accepted (A. *r* = -0.40; B. *r* = -0.48), while a weak correlation was found for the conditions fair accepted (A. *r* = 0.04) and unfair rejected (B. *r* = 0.14). *Note.* for both interaction effects, the difference between the two slopes remains significant, after removal of the blue and red data point on the right side of the plot. D. striatum = dorsal striatum

## Appendix A

### Movie clips

Before the proposal, a movie clip was played in which participants observed their opponent sitting behind a computer screen from a front left-side view. The role of the opponent was played by a female student of the same age as the participants. In 18 of the 24 movie clips, participants observed their opponent moving the mouse with her right hand in order to propose a division. In the other six movie clips, participants observed a similar action in addition to some naturally occurring behavior, such as taking a sip of water or leaning back. Notably, the opponent briefly looks into the camera during the movie clip of the second trial. This action is supposed to signal that the opponent is aware of the fact that she is videotaped. The movie clips were played serially and had a duration of six seconds.

**Table 5:** Overview of the order of the movie clips

Movie clip nr.	Movie clip content
1	Proposing a decision
2	Proposing a decision + Looking briefly into the camera
3	Proposing a decision
4	Proposing a decision
5	Proposing a decision
6	Proposing a decision
7	Proposing a decision
8	Proposing a decision
9	Proposing a decision
10	Proposing a decision
11	Proposing a decision + Scratching the head
12	Proposing a decision
13	Proposing a decision
14	Proposing a decision
15	Proposing a decision + Positioning the left hand under the chin
16	Proposing a decision
17	Proposing a decision
18	Proposing a decision + Taking a sip of water
19	Proposing a decision
20	Proposing a decision + Leaning backwards
21	Proposing a decision
22	Proposing a decision
23	Proposing a decision + Positioning the left hand under the chin
24	Proposing a decision

## Appendix B

### Overview of the full fMRI session

The full fMRI session consisted of four tasks, resting state and an anatomical scan. The following tasks/scans were presented in consecutive order: emotional face-matching task (Hariri et al., 2002), mood (worry) induction paradigm (Paulesu et al., 2010), anatomical scan, resting state, interoceptive sensitivity task (Pollatos, Herbert, Matthias, & Schandry, 2007) and Ultimatum Game (Sanfey et al., 2003). The total duration of the fMRI session was approximately 60 minutes. The order was fixed and identical for all participants.

## Appendix C

### Preprocessing steps

First, structural as well as functional images were reoriented parallel to the AC-PC plane. Second, functional images were realigned to the first image using rigid body transformations, and the mean EPI image, created during this step, was coregistered to the anatomical T1 image. Third, structural images were corrected for bias field inhomogeneities, registered using linear transformations and segmented into grey matter (GM), white matter (WM), and cerebrospinal fluid (CSF; MNI template space). Fourth, we used DARTEL (Diffeomorphic Anatomical Registration through Exponentiated Lie algebra toolbox; Ashburner, 2007) to create a customized group template to increase the accuracy of intersubject alignment. Individual GM and WM tissue segments were iteratively aligned to the group template in order to acquire individual deformation flow fields. Fifth, the coregistered functional images were normalized to MNI space using the customized group template and individual deformation flow fields. Furthermore, images were resampled to 2 mm^3^ isotropic voxels and smoothed with an 8-mm full-width at half-maximum (FWHM) Gaussian kernel.

## Appendix E

Results related to the proposal, decision and outcome condition in association with neuroticism thresholded at *p* < .05 FWE cluster-level extent using an initial threshold of *p* < .001 and/or *p* < .01

**Table 6:** Interaction effects between neuroticism and the proposal, decision and outcome condition thresholded at *p* < .05 FWE cluster level extent using an initial threshold of *p* < .001 and/or *p* < .01

Cluster	Cluster size	*Z* value	Coordinates		
			*x*	*y*	*z*
Decision: unfair accept > fair accept * neuroticism (*p* < .01 uncorrected and *k* > 316)					
Cerebellum Vermis	831	-2.58	-16	-34	-20
Putamen Insula	658	-2.58	34	-22	20
Decision: unfair accept > unfair reject * neuroticism (*p* < .01 uncorrected and *k* > 316)					
Cerebellum Vermis	427	-2.58	4	-48	-22
Putamen Insula	702	-2.58	14	-14	12
Outcome: unfair accept > fair accept * neuroticism (*p* < .001 uncorrected and *k* > 102)					
Lingual gyrus Cerebellum	119	-3.11	-28	-86	-12
Parahippocampal gyrus Cerebellum	106	-3.10	-10	-26	-18
Middle temporal gyrus	325	-3.10	-40	-56	14
Supplementary motor area Precentral gyrus	185	-3.10	12	-18	70
Outcome: unfair accept > unfair reject * neuroticism (*p* < .001 uncorrected and *k* > 102)					
Cerebellum Vermis	164	-3.10	6	-24	-10
Parahippocampal gyrus Cerebellum	123	-.3.10	-8	-36	-20
Outcome: unfair accept > fair accept * neuroticism (*p* < .01 uncorrected and *k* > 316)					
Lingual gyrus Fusiform gyrus Parahippocampal gyrus Cerebellum	853	-2.58	-8	-22	-4
Inferior occipital gyrus Middle occipital gyrus Lingual gyrus Cerebellum	501	-2.58	-10	-88	-6
Inferior occipital gyrus Middle occipital gyrus Lingual gyrus Calcarine sulcus	393	-2.58	24	-84	4
Middle temporal gyrus	590	-2.58	-40	-60	6
Precentral gyrus	360	-2.58	-38	0	44
Supplementary motor area Paracentral lobule	499	-2.58	-8	-4	70
Outcome: unfair accept > unfair reject * neuroticism (*p* < .01 uncorrected and *k* > 316)					
Cerebellum Vermis	1111	-2.58	0	-36	-14

Peak activations with corresponding *Z* values of brain regions, which showed differential activation for the contrasts decision: unfair accept > fair accept * neuroticism, decision: unfair accept > unfair reject * neuroticism, outcome: unfair accept > fair accept * neuroticism and outcome: unfair accept > unfair reject * neuroticism. To correct for multiple comparisons, resulting brain images were thresholded at *p* < .05 FWE cluster-level extent using an initial threshold of *p* < .001 and/or *p* < .01 uncorrected and a cluster size of *k* > 102 and/or *k* > 316, respectively. The extent threshold (*k*) was obtained via Monte Carlo simulation (*3dClustSim*, AlphaSim, implemented in AFNI, 10,000 iterations). Results for the proposal and decision condition, thresholded at *p* < .05 FWE cluster-level extent using an initial threshold of *p* < .001 uncorrected (and a cluster size of *k* > 102), can be found in the main text
